# Multifunctional Receptor Stabilin-1 in Homeostasis and Disease

**DOI:** 10.1100/tsw.2010.189

**Published:** 2010-10-12

**Authors:** Julia Kzhyshkowska

**Affiliations:** ^1^ Department of Dermatology, University Medical Centre Mannheim, Ruprecht-Karls University of Heidelberg, Mannheim, Germany; ^2^ Institute of General Pathology and Pathophysiology, Russian Academy of Medical Sciences, Moscow, Russia

**Keywords:** macrophage, endothelial cell, endocytosis, phagocytosis, secretion, transcytosis, cell adhesion, GGAs, SPARC, placental lactogen, chitinase-like protein, SI-CLP, trans-Golgi network

## Abstract

The multifunctional scavenger receptor stabilin-1 (STAB1, FEEL-1, CLEVER-1, KIAA0246) is expressed on tissue macrophages and sinusoidal endothelial cells in healthy organisms, and its expression on both macrophages and different subtypes of endothelial cells is induced during chronic inflammation and tumor progression. Stabilin-1 is a type-1 transmembrane receptor that mediates endocytic and phagocytic clearance of “unwanted-self” components, intracellular sorting of the endogenously synthesized chitinase-like protein SI-CLP, and transcytosis of the growth hormone family member placental lactogen. The central sorting station for stabilin-1 trafficking seems to be the trans-Golgi network (TGN). Transport of stabilin-1 in the TGN requires interaction with GGA adaptors that bind to the classical DDSLL motif and a novel acidic cluster in its cytoplasmic tail. Degradation of stabilin-1 seems to depend on the interaction with sorting nexin 17. However, the mechanisms keeping stabilin-1 on the cell surface remain to be identified. This issue deserves specific attention due to the growing amount of data indicating that function of stabilin-1 in cell adhesion events is essential for inflammation and metastasis. Taking into consideration the complexity of stabilin-1—mediated processes, investigation of stabilin-1 functions in the animal models, as well as mathematic modeling of intracellular trafficking and extracellular contact, would enable prediction of stabilin-1 behavior in complex biological systems and would open perspectives for therapeutic targeting of stabilin-1 pathways in chronic inflammation and carcinogenesis.

## STABILIN-1 — FIRST IDENTIFIED MEMBER OF THE STABILIN FAMILY

The first member of the stabilin family, human stabilin-1, was identified in 1991 as a specific histological marker for noncontinuous sinusoidal endothelial cells in the human spleen and was named MS-1 antigen[1]. The monoclonal antibody MS-1 raised against the human spleen was selected by immunohistological reactivity with cadherin-negative noncontinuous endothelium. Applying classical biochemical methodology, the MS-1 antigen was purified and analyzed by MALDI-TOF. The MALDI-TOF MS (matrix-assisted laser desorption ionization – time of flight mass spectrometry) chromatogram allowed identification of partial cDNA, and a full-length cDNA of about 8 kb was generated using 3' and 5'-RACE (rapid amplification of cDNA ends). The protein encoded by the full-length cDNA had a predicted molecular weight of 280 kDa and was named stabilin-1. Northern blot analysis showed a selective expression of the stabilin-1 cDNA in tissues with noncontinuous endothelial cells (spleen, liver, lymph nodes) and in alternatively activated macrophages obtained by *in vitro* differentiation of human monocytes in the presence of interleukin (IL)-4 and dexamethasone. These data correlated with the results of extensive immunochemical screening performed earlier using MS-1 monoclonal antibodies[1,2,3]. Final confirmation for the identity of the MS-1 antigen and stabilin-1 has been obtained with the help of the new rabbit polyclonal antibody F4, generated against the recombinant glutathione S-transferase (GST)–tagged cytoplasmic tail of stabilin-1. Both F4 and MS-1 recognized an identical pattern in double immunofluorescent analysis of alternatively activated human macrophages[4]. Bioinformatic analysis revealed that stabilin-1 is a type-1 transmembrane protein with a large extracellular part containing clusters of epidermal growth factor (EGF)–like domains, seven fasciclin domains, and one X-link domain (Fig. 1).

In parallel to the stabilin-1 cDNA, a homologous protein – stabilin-2 – was cloned that appeared to be a true hyaluronan receptor of the hepatic sinusoidal endothelial cells[4,5]. Stabilin-1 and -2 are 55% identical on the protein level, but show no significant DNA homology. The murine stabilins have also been cloned by us[4]. The homology between human and mouse is 86% for stabilin-1 and 79% for stabilin-2, which indicates the high evolutionary conservation of these two proteins. Interestingly the C-terminal parts of two stabilins exhibit the major differences. Stabilin-2 contains a classical tyrosine-based endosomal sorting signal, while stabilin-1 contains a special dileucine-based sorting motif that was functionally investigated by us[4,6].

Later, other laboratories independently identified stabilin-1 and named it FEEL-1 (fasciclin, EGF-like, laminin-type EGF-like, and link domain-containing scavenger receptor-1[7,8]) and CLEVER-1 (common lymphatic endothelial and vascular endothelial receptor-1[9]). Adachi and Tsujimoto searched for the novel scavenger receptor on endothelial cells and identified FEEL-1 using the expression cloning technique[8]. CLEVER-1 has been found as an antigen recognized by the mouse monoclonal antibodies raised against isolated efferent lymphatic vessels of human lymph nodes, and blocking adhesion of lymphocytes to high endothelial venules (HEV) and lymphatic endothelium *in vitro*. Purification of the CLEVER-1 antigen by affinity chromatography and mass spectrometric analysis of peptides obtained by trypsin digestion revealed the identity of CLEVER-1 and stabilin-1[9], and was later confirmed by costaining of different tissues with CLEVER-1 and rabbit polyclonal anti–stabilin-1 antibodies (our unpublished observations).

## EXPRESSION OF STABILIN-1 IN HEALTH AND PATHOLOGY

Extensive analysis of human tissues performed using MS-1 antibody indicated that the two major cell types expressing stabilin-1 *in vivo* are tissue macrophages and noncontinuous endothelial cells (ECs) (reviewed in [10]). Human sinusoidal ECs in spleen, liver, adrenal cortex, lymph nodes, and bone marrow express high amounts of stabilin-1[1,11,12,13,14]. In the lymph nodes, stabilin-1 is expressed not only on sinusoidal ECs, but also on sinusoidal macrophages; however, stabilin-1 is absent from Kupffer cells – liver macrophages located in close proximity to liver sinusoidal ECs[13]. Sinusoidal ECs form noncontinuous endothelium, while blood vessels in healthy organism are constituted of continuous endothelium. Rapidly growing blood vessels pass through the temporary noncontinuous state during angiogenesis. We and others demonstrated that stabilin-1 is expressed on blood vessels in various angiogenic conditions, including wound healing, tumor vascularization, and chronic inflammation of the skin, such as psoriasis[2,15].

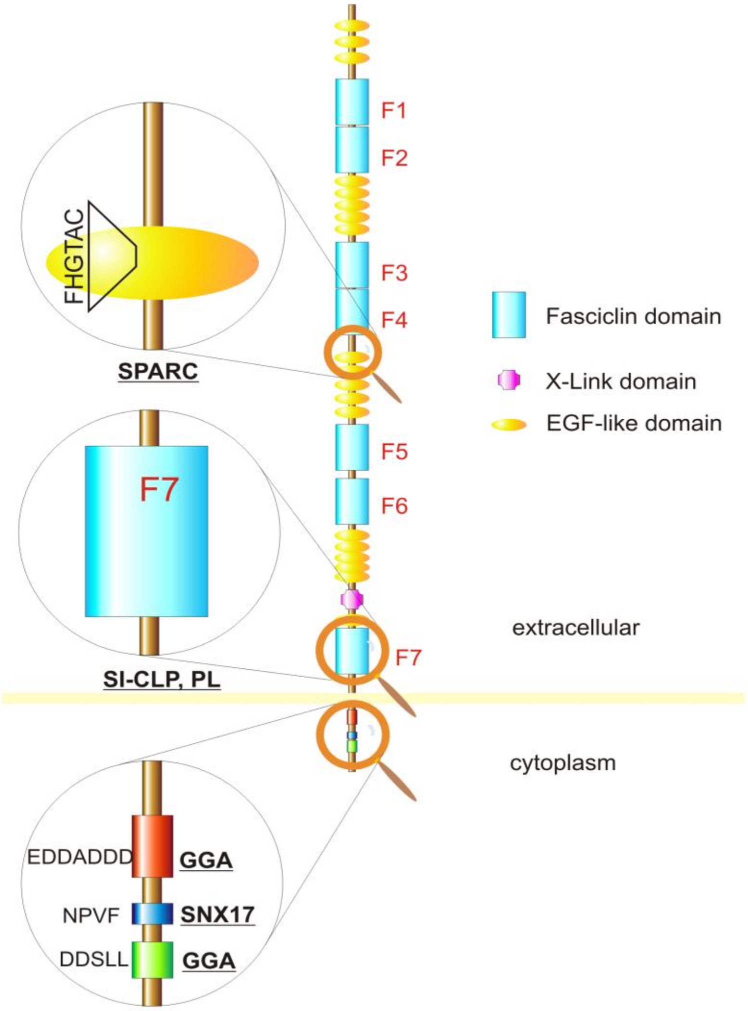

**FIGURE 1.** Stabilin-1 domain organization, binding sites for the ligands, and sorting adaptors. The large extracellular fragment of stabilin-1 is composed of seven fasciclin domains (blue cylinders F1–F7) and multiple EGF-like domains (yellow ovals). The X-link domain is shown in pink. SPARC (secreted protein acidic and rich in cysteine) binds to the EGF-like domain located between F4 and F5; the FHGTAC sequence was identified as SPARC-binding peptide in phage display. The chitinase-like protein SI-CLP and placental lactogen bind to F7. The short cytoplasmic tail contains a classical DDSLL motif and novel acidic cluster EDDADDD interacting with intracellular sorting adaptors – GGA1, GGA2, and GGA3L, as well as the NPVF site interacting with sorting nexin 17 (SNX17). Names of proteins that bind stabilin-1 are underlined.

There are some evidences about the presence of stabilin-1 on lymphatic endothelium. Searching with the MS-1 antibody for the expression of stabilin-1 on lymphatic vessels in different organs revealed a very weak signal only in healthy human skin[16]. The group of Jalkanen demonstrated that α-CLEVER-1 antibody detects stabilin-1 on lymphatic vessels in various tissues and in various physiological conditions[9,15,17]. Prevo et al. used a panel of antibodies against various domains of stabilin-1 to carefully examine localization of stabilin-1 on lymphatic vessels[11]. The anti–CLEVER-1 3-372 monoclonal antibody weakly recognized stabilin-1 on lymphatic vessels in frozen sections of human skin. However, antibodies generated against the last 14 amino acids of the stabilin-1 cytoplasmic tail and against the X-link domain did not recognize stabilin-1 in lymphatic vascular endothelial hyaluronan receptor-1 (LYVE-1)–positive vessels in various organs, including stomach and colon. The discrepancies between different studies can be explained by variations in experimental procedures, such as fixation or staining conditions, or by the recognition by various antibodies distinct epitopes of stabilin-1.

Stabilin-1 expression on macrophages *in vitro* requires the presence of glucocorticoids (reviewed in [10]). In human monocyte-derived macrophages, the stabilin-1 protein can be detected in cultures generated under the stimulation by IL-4 in combination with dexamethasone or by dexamethasone alone (alternatively activated macrophages), while interferon (IFN)-γ has a negative effect on stabilin-1 expression[2,4,6]. *In vivo*, stabilin-1 is used as a specific marker for alternatively activated macrophages, including subpopulations of tumor-associated macrophages (TAM)[18,19,20]. We found that in murine tumor models and in an excision wound healing model, stabilin-1 is expressed together with LYVE-1 on a subset of CD11b(+), F4/80(+) tissue macrophages. Stabilin-1+ macrophages have been found predominantly on the periphery of B16F1 melanoma, while only single stabilin-1+ macrophages have been detected inside of the tumor mass. Preferential localization of stabilin-1+ macrophages on the periphery of the melanoma might indicate their protective role restricting growth of melanoma[20]. Our most recent data indicate that stabilin-1 is also abundantly expressed on TAM in the animal model for breast cancer[21]. The role of stabilin-1+ macrophages in tumor progression needs further investigation in stabilin-1 knock-out mice.

Increased numbers of stabilin-1+ and CD163+ duodenal macrophages have been identified immunohistochemically in patients with Whipple's disease, a chronic multisystemic infection caused by *Tropheryma whipplei*[22]. The authors suggested that lack of excessive local inflammation and alternative activation of macrophages, triggered partially by the *T. whipplei* itself, explains the hallmark of Whipple's disease: invasion of macrophages that are not able to clear *T. whipplei* in the intestinal mucosa[22]. However, the specific contribution of stabilin-1 in the pathogenesis Whipple's disease is an unexplored issue.

The most surprising recent finding was identification of stabilin-1 on the circulation monocytes in the blood of patients with familial hypercholesterolemia (FH)[23]. FH is characterized by raised plasma low-density lipoprotein (LDL) cholesterol levels, leading to accelerated atherosclerosis and increased risk of premature coronary heart disease. FH results from defects in the hepatic clearance of LDL via the LDL-receptor pathway, commonly caused by a loss-of-function mutation in the LDL-receptor gene (LDLR) or by a mutation in the gene encoding apolipoprotein B (APOB)[24]. Significant differences between monocyte subsets in FH patients and in healthy donors have been characterized using flow cytometry, scanning microscopy, and functional tests, such as endocytosis, phagocytosis, and adhesion assay. FH-CD14(+)CD16(+) monocytes were positive for stabilin-1, as well as for CD68 and CD11c. This monocyte subpopulation showed increased CD36-mediated uptake of oxLDL, and adherence to activated ECs in response to oxLDL and nLDL stimulation[23]. Investigation of the role of stabilin-1 in these two processes requires generation of α-stabilin-1 blocking antibodies. This is a difficult task since stabilin-1 has a large extracellular domain with repetitive structures and a single antibody is unlikely to block stabilin-1 interactions completely.

The expression profile of stabilin-1 highlights its presence on the professional scavenging cells. Their major common function is clearance of blood or lymphatic circulation (sinusoidal cells) or extracellular space in tissues (tissue macrophages) from nonself and unwanted-self components. This clearance activity is essential for the homeostatic balance in tissues and in the whole organism. Expression of the type-1 transmembrane protein stabilin-1 on both cell types suggested its function as a scavenger receptor. Detailed investigation of the intracellular trafficking pathways of stabilin-1 and identification of its ligands is described in below.

## INTRACELLULAR DISTRIBUTION AND INTERACTION WITH SORTING ADAPTORS

Structurally, stabilin-1 is a type-1 transmembrane receptor with a short and unique cytoplasmic tail (see Fig. 1). However, its function as a surface receptor was questionable for a long time, since all histological data indicated its intracellular vesicular localization both in macrophages and in sinusoidal ECs. FACS

analysis of enhanced sensitivity provided the first indication that stabilin-1 is exposed on the cell surface of alternatively activated human macrophages[6]. Detailed investigation of stabilin-1 localization in various vesicular compartments in macrophages showed that stabilin-1 has multiple functions in these immunoregulatory cells. The first pathway was classical for an endocytic receptor. Stabilin-1 was predominantly localized in early endosome antigen 1 (EEA-1)–positive early endosomes, its minor portion was found in late endosomes, while a portion of stabilin-1 was recycled to the cell surface (Fig. 2).

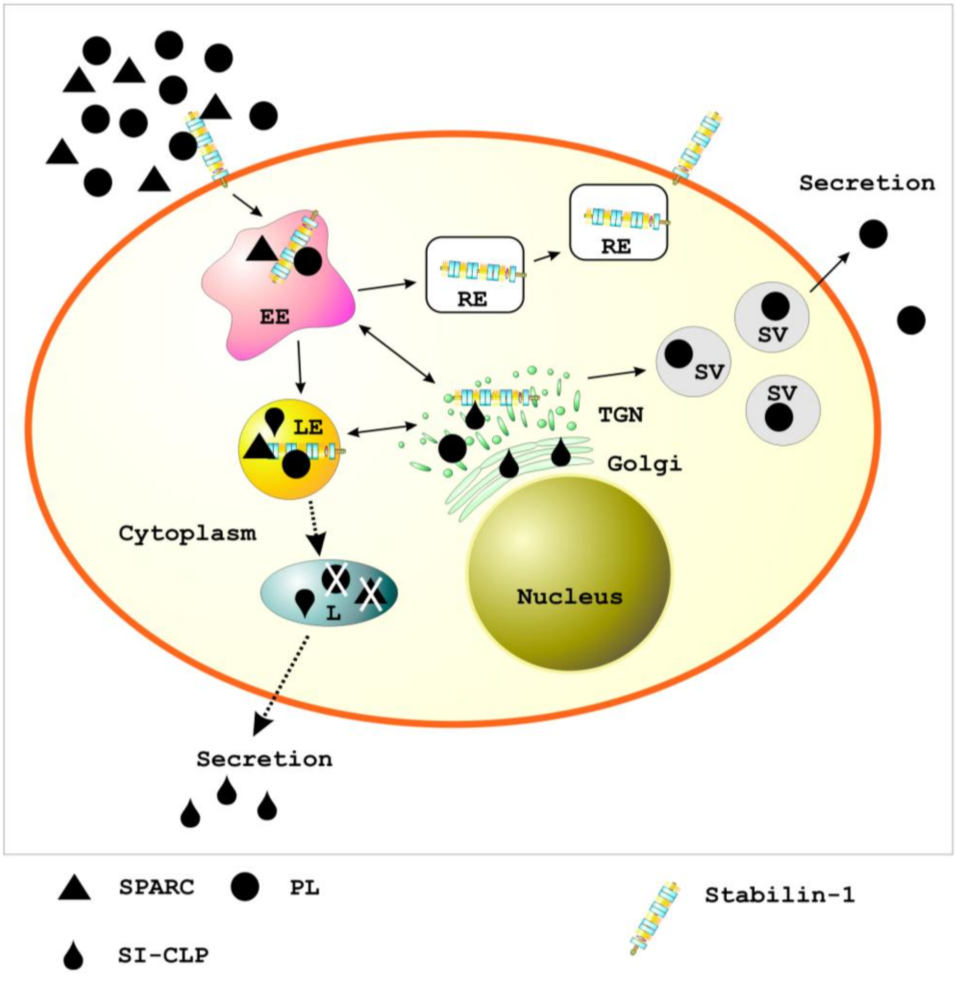

**FIGURE 2**. Complex trafficking pathways of stabilin-1 and its ligands. Stabilin-1 recognizes extracellular endocytic ligands SPARC (shown as a filled triangles) and placental lactogen (PL) (shown as a filled circles), as well as newly synthesized intracellular sorting ligand SI-CLP (filled drops). Upon binding to the surface-expressed stabilin-1, endocytic ligands are internalized and delivered to early-sorting endosomes (EE). A portion of the ligand-free receptor can recycle back to the cell surface via recycling endosomes (RE). Stabilin-1 targets both SPARC and PL to late endosomes (LE), and consequently for the degradation in lysosomes (L). Part of PL escapes degradation and is delivered by stabilin-1 to the trans-Golgi network (TGN). In the TGN, PL dissociates from stabilin-1, is further transported to the new type of storage vesicles (SV), and can be secreted to the extracellular space. Stabilin-1 is also involved in the intracellular sorting process; it shuttles between endosomes and TGN. We propose a model whereby newly synthesized SI-CLP is recognized by stabilin-1 in the late Golgi compartment and delivered LE. In LE, SI-CLP dissociates from stabilin-1, is transported to the lysosomes, which further can undergo stimuli-dependent secretion (modified from Kzhyshkowska et al.[86]).

Unexpectedly, we found that stabilin-1 shuttles between early endosomes and the trans-Golgi network (TGN) in human macrophages[6]. In rat liver sinusoidal ECs, stabilin-1 was found in the endocytic pathway, but not in the endosome-TGN sorting pathway, suggesting that the intracellular sorting function of stabilin-1 is specific for macrophages[12]. Searching for the intracellular sorting machinery used by stabilin-1, we found that the DDSLL motif in its cytoplasmic tail interacts with sorting adaptors – GGAs. GGAs (Golgi-localized, γ-ear–containing, Arf [ADP-ribosylation factor]-binding proteins) function as clathrin adaptors for intracellular sorting of mannose-6 phosphate receptors (MPRs), sortillin, and others[25]. In the case of CI (cation independent)-MPR, GGAs mediate its shuttling between early endosomes and TGN, the pathways used by CI-MPR to deliver newly synthesized lysosomal hydrolases from the biosynthetic pathway to the lysosomes in a safe manner. The interaction of GGAs with the cytoplasmic tail of stabilin-1 was not restricted to the binding to the classical DDSLL motif, but was also mediated by a novel acidic cluster, EDDADDD, identified by us. We showed that both DDSLL and EDDADDD sites have to be deleted in the cytoplasmic tail of stabilin-1 in order to completely abrogate its interactions with GGAs. However, surface exposure of recombinant stabilin-1 in stably transfected CHO cells was not affected by the deletion of both motifs indicating that stabilin-1 delivery to the cell surface does not requires GGAs[26].

The second function of CI-MPR is receptor-mediated endocytosis of insulin-like growth factor-II; however, this pathway does not require GGAs[27]. Similarity in intracellular routing between stabilin-1 and CI-MPR was intriguing. Given that CI-MPR is ubiquitously expressed, we suggested that stabilin-1 is a first macrophage–specific receptor combining endocytic and intracellular sorting functions, and our research was focused on the identification of ligands that are internalized and sorted by stabilin-1 in human macrophages.

The recent study of Adachi and Tsujimoto identified sorting nexin 17 (SNX17) as a trafficking adaptor of stabilin-1 in ECs[28]. The NPVF motif in the cytoplasmic tail of stabilin-1 was required for the interaction with SNX17. SNX17 seems to be responsible for the targeting of stabilin-1 for degradation, at least in ECs. Whether SNX17 is essential for stabilin-1 trafficking in macrophages remains to be identified.

## STABILIN-1 IS AN ENDOCYTIC RECEPTOR FOR “SELF” LIGANDS

Specific expression of stabilin-1 on professional clearance cells prompted us to investigate its function as a scavenger receptor. Both macrophages and sinusoidal endothelial cells (SEC) express a broad range of classical scavenger receptors that internalize and target for the degradation nonself and “unwanted-self” molecules and particles[29,30,31]. Originally scavenger receptors were defined by their ability to endocytose modified lipoproteins such as oxLDL and its analogue acLDL (reviewed in [32]). A characteristic feature of scavenger receptors is their overlapping ligand repertoire that is essential for the efficient and safe function of macrophages during resolution of inflammation and homeostatic tissue turnover[33,34]. We used CHO cells lacking endogenous scavenger receptors for acLDL (including stabilin-1) for the ectopic stable expression of stabilin-1 in order to analyze its specific contribution in the uptake of acLDL[35]. FACS analysis and confocal microscopy revealed that stably expressed stabilin-1 functions as an efficient endocytic receptor for acLDL in CHO cells. Similar results were obtained by other groups in CHO[8] and HeLa cells[11] transiently transfected with stabilin-1. Phosphatidylinositol 3-kinases (PI3-kinases, PI3Ks) are critical regulators of the endocytosis mediated by scavenger receptors. Class III PI3K activity is required for the production of phosphatidylinositol-3-phosphate (PtdIns(3)P) that is incorporated in the membranes of early endosomes and is recognized by FYVE-domain–containing proteins and sorting nexins. (The FYVE-domain is named after the four cysteine-rich proteins: Fab 1 [yeast orthologue of PIKfyve], YOTB, Vac 1 [vesicle transport protein], and EEA1, in which it has been found). This recognition event is required for Rab-5–dependent endosomal fusion and membrane trafficking. The fact that stabilin-1–mediated transport of acLDL along the lysosomal-targeted endocytic pathway requires PI3K activity further confirmed that stabilin-1 uses classical endocytic pathways for the targeting of its extracellular cargo for degradation[35,36].

The homeostatic function of stabilin-1 was initially suggested due to its unusual function – internalization and clearance of the universal regulator of tissue turnover – SPARC[37]. The glycoprotein SPARC (secreted protein acidic and rich in cysteine, also known as osteonectin and BM-40) is a soluble, nonstructural component of the extracellular matrix (ECM), identified over 2 decades ago (reviewed in [38]). SPARC is a crucial regulator of developmental processes, tissue remodeling, angiogenesis, wound healing, tumor progression, obesity, and diabetes[39,40,41,42]. Its multiple biological functions include active modulation of ECM organization, binding of growth factors, and induction of an antiadhesive state of different cell types[43,44,45]. Despite the long history of the functional and biochemical analysis of SPARC, the cellular receptor(s) mediating responses to SPARC, as well as regulating its activity and extracellular concentration, remained unknown until our identification of stabilin-1 as a scavenger receptor for SPARC. In our recent collaborative work, we identified stabilin-1 as an interacting partner of SPARC using phage display technology[37]. By *in vitro* binding assay, we demonstrated that stabilin-1 interacts with SPARC through the extracellular EGF-like domain containing the sequence FHGTAC (see Fig. 1). In stably transfected CHO cells, stabilin-1 efficiently endocytosed SPARC, whereas acLDL was competing with SPARC internalization in a concentration-dependent manner. Only stabilin-1–positive macrophages, stimulated by IL-4 and dexamathasone, but not stabilin-1–negative macrophages, were able to internalize SPARC efficiently, transport it to the endocytic pathway, and degrade it. Both transport pathway and kinetics of degradation were similar for SPARC and acLDL[37]. We hypothesize that, via uptake and degradation of SPARC, stabilin-1–positive macrophages can actively regulate extracellular concentrations of SPARC and adjust them to the actual physiological needs of the tissue. In a pathological situation, SPARC activity can be controlled by macrophages, which have flexible phenotype and constitute essential innate immune cells that respond to the tissue damage and cellular transformation. Thus, diverse effects of SPARC observed in various animal models for carcinogenesis and healing might depend on the phenotype of local macrophages. For example, it has been published that SPARC reduces tumor activity in breast cancer[46]; however, it promotes progression of malignant melanoma[47]. We can hypothesize that stabilin-1 expressed on TAM-infiltrating breast tumors[21] supports tumor growth, while stabilin-1 expressed on the TAM localized at the periphery of a malignant melanoma[20] will rather restrict tumor progression. The role of stabilin-1 in tumor progression is currently under investigation in knock-out animal models in our laboratory.

Alternatively, activated (M2) macrophages[48] regulate immune responses and tissue remodeling, not only in adult tissues, but also in the placenta. Stabilin-1 is abundantly expressed both in decidual macrophages and in Hofbauer cells. We applied yeast two-hybrid screening using human placental cDNA library, and succeeded with the identification of the developmental hormone placental lactogen (PL) as a novel ligand of stabilin-1[49]. PL belongs to the growth hormone/prolactin (GH/PRL) family of polypeptide regulators of pregnancy, postnatal growth, and lactogenesis, acting both as a circulating hormone and as a local paracrine/autocrine factor[50]. Human PL (hPL) has a 96% similarity to GH and is produced by cytotrophoblast and syncytiotrophoblast cells. We found that similarly to SPARC, PL was efficiently endocytosed by stabilin-1 in the stably transfected CHO cells and stabilin-1–positive primary human monocyte-derived macrophages stimulated with IL-4 and dexamethasone. Moreover, we were able to demonstrate the uptake of the fluorescently labeled PL by macrophages isolated from villi of human placenta/stabilin-1–positive Hofbauer cells that are localized in close proximity to the trophoblast cell producing a high amount of PL[49]. Surprisingly, only part of PL has been degraded in stabilin-1–positive macrophages, while part has been sorted into the alternative secretory pathway described below.

Since the cytoplasmic tail of stabilin-1 does not have a classical motif for the interaction with a clathrin adaptor AP-2 that mediates internalization of numerous of receptors, including stabilin-2[12], we explored the role of GGAs in the internalization of stabilin-1 upon its binding to the extracellular ligands. Using CHO cells stably expressing recombinant stabilin-1 with deletion either of DDSLL or EDDADDD, or both, we found that all mutants internalize acLDL, SPARC, and PL, as well as anti–stabilin-1 monoclonal antibody MS-1, with similar efficiency, indicating that GGAs are not involved in the stabilin-1 endocytosis. The sorting machinery that is responsible for the internalization of stabilin-1 remains to be identified.

## ENDOCYTIC LIGANDS OF STABILIN-2 AND STABILIN-1

Stabilin-2 (FEEL2; HARE/hyaluronan receptor for endocytosis) has a very similar extracellular domain organization with stabilin-1, but differs from stabilin-1 by its absence on macrophages, by the absence of GGA binding sites in its cytoplasmic tail, and by performing a solely classical endocytic function[4,51]. Being originally identified as a clearance receptor for hyaluronan expressed on liver sinusoidal ECs, stabilin-2 is known now as a scavenger receptor for modified unwanted-self products with broad specificity[4,5,7,12,52]. Stabilin-2 ligands include hyaluronic acid (HA), AGE-modified BSA (advanced glycation end product–conjugated bovine serum albumin), formaldehyde-treated BSA, collagen N-terminal propeptides, chondroitin sulfates, and high- and low-molecular-weight heparins[7,12,53,54,55]. Out of these ligands, stabilin-1 is known to express weak binding activity only for AGE-BSA, however physiological significance of this *in vitro* interaction remains unclear[7]. Stabilin-1 has an extracellular X-link domain that is known to function as a HA-binding site in several receptors, including stabilin-2, CD44, and LYVE-1. However, all attempts using various *in vitro* systems and various sources of HA failed to detect HA binding to stabilin-1[11,12,15,56]. Chondroitin sulfates, as well as high- and low-molecular-weight heparins remain to be tested as ligands for stabilin-1. Thus, currently, the only proven common ligand shared by stabilin-1 and stabilin-2 is acLDL([35,54] and our unpublished data).

## INTRACELLULAR SORTING FUNCTION OF STABILIN-1: TRANSPORT TO THE LYSOSOMAL SECRETORY PATHWAY AND TRANSCYTOSIS

Shuttling of stabilin-1 between endosomal and biosynthetic compartments in macrophages and its interaction with GGA adaptors indicated that stabilin-1 functions as sorting receptor for the endogenous ligands. Using yeast two-hybrid screening, we identified an intracellular ligand for stabilin-1 – a novel chitinase-like protein SI-CLP (stabilin-interacting chitinase-like protein)[57].

SI-CLP is a member of the mammalian family of Glyco_18 domain–containing proteins, comprising true chitinolytic enzymes as well as secreted chitinase-like proteins lacking enzymatic activity (Fig. 3). Two members of this family, chitotriosidase and AMCase are able to bind chitin via the C-terminal chitin-binding domain and hydrolyze polysaccharides[58,59,60,61,62]. Hydrolytic activity of chitotriosidase and AMCase indicates their conservative function in host defense against chitin-containing pathogens[62,63] and enzymatic activity of AMCase contributes to asthma progression[64]. Enzymatically silent Glyco_18 domain–containing chitinase-like proteins do not bind chitin, but rather act as soluble mediators and might be involved in tumor progression[65,66,67,68,69,70] and disorders characterized by chronic inflammation and ECM remodeling, such as rheumatoid arthritis[71], inflammatory bowel disease[72], hepatic fibrosis and cirrhosis[73,74], and systemic sclerosis[75]. Crucial biological functions were assigned to mammalian chitinase-like proteins including mediation of cell differentiation, proliferation activation, migration, and adhesion[76,77,78,79,80,81], however, no receptors for chitinase-like proteins have been found until our identification of stabilin-1/SI-CLP as a ligand-receptor pair[57].

Expression analysis of SI-CLP performed by real-time PCR and Western blotting using novel rat monoclonal antibody 1C11 revealed that production of SI-CLP is up-regulated in stabilin-1–positive primary human macrophages[57]. Using immunofluorescent/confocal microscopy analysis, we further demonstrated that major sites of SI-CLP intracellular localization are secretory lysosomes. Other mammalian Glyco_18 domain–containing proteins are also sorted into lysosomes, however the lack of N-glycosylation typical for lysosomal enzymes indicates that their lysosomal routing is different from a classical MPR-mediated one[82].

Several facts support the point of view that stabilin-1 acts as a sorting receptor for SI-CLP, at least in stabilin-1–positive macrophages. The extracellular domain of the type-1 transmembrane receptor can be exposed to late Golgi compartments for the recognition of newly synthesized cargo protein, and we found that SI-CLP binds to the extracellular fasciclin 7 (F7) domain of stabilin-1[57] (Fig. 1). Stabilin-1

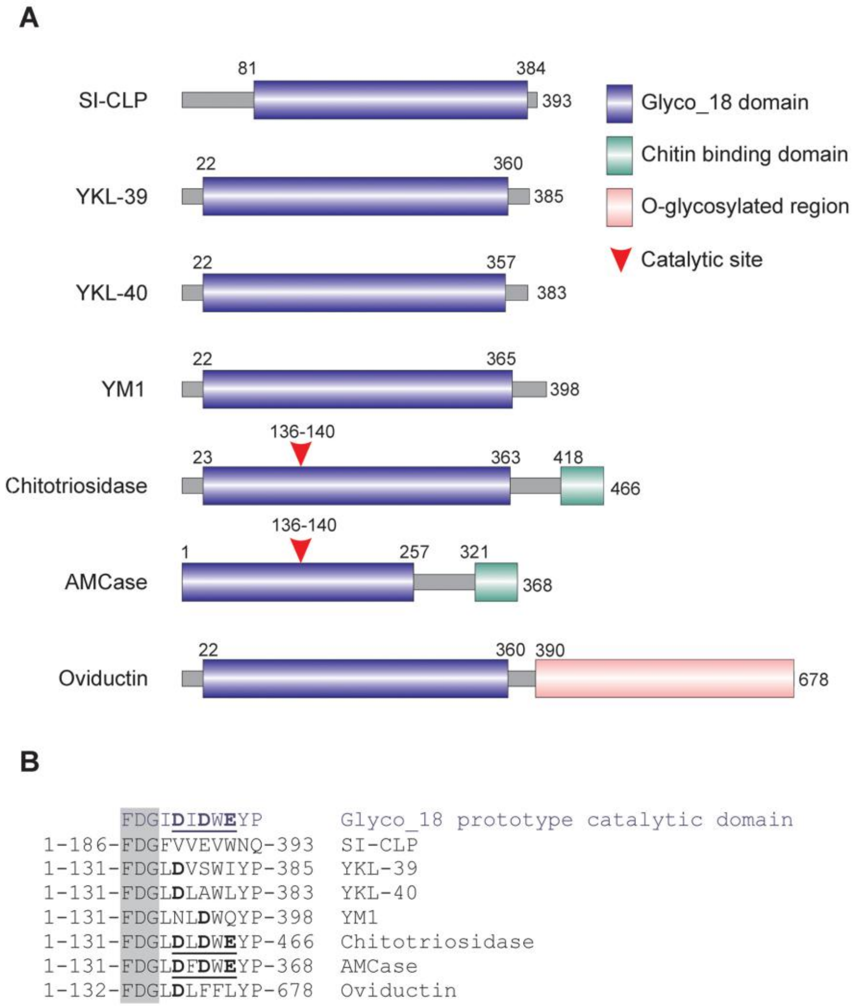

**FIGURE 3.** SI-CLP is a novel member of Glyco_18 domain–containing human chitinases and chitinase-like proteins. (A) Schematic presentation of mammalian Glyco_18 domain–containing proteins. (B) Critical amino acid in catalytic sites. The characteristic FDG sequence preceding the catalytic motif is shown in shadowed box. Catalytic amino acids are shown in bold. Complete active catalytic motifs are underlined. This research was originally published in Kzhyshkowska et al.[57]. © The American Society of Hematology.

colocalization with SI-CLP in the dynamic TGN is transient, and SI-CLP seems to dissociate from stabilin-1 due to low pH in late endosomes, as it was established for many other classical receptor-ligand interactions. Being transiently overexpressed, stabilin-1 mediates relocalization of recombinant SI-CLP-FLAG in H1299 cells that lack the endogenous lysosomal sorting machinery for this protein[57]. Deletion of GGA-binding sites in the cytoplasmic tail for stabilin-1 abrogates stabilin-1–mediated sorting of SI-CLP in H1299, indicating also that both classical DDSLL as well as novel EDDADDD GGA binding sites are essential for stabilin-1–mediated intracellular sorting[26]. And, finally, knock-down of stabilin-1 in macrophages results in impaired lysosomal sorting of SI-CLP and its accumulation in perinuclear TGN-like structures[57].

TGN-related sorting activities of stabilin-1 are not restricted to the transport of endogenously synthesized SI-CLP. The unique function of stabilin-1 is targeting of the internalized ligand-PL to the TGN[49]. As it has been described in the paragraph about endocytic functions of stabilin-1, endocytosed PL is only partially degraded in lysosomes of stabilin-1–positive macrophages. Part of PL escapes degradation and is delivered to the novel PL+ storage vesicles negative for endosomal/lysosomal markers and lacking stabilin-1. During formation, stabilin-1+ PL+ vesicles transiently accumulate in the TGN, and the most intensive accumulation of PL in TGN was detected between 1 and 3 h after endocytosis. The PL+ stabilin-1– storage vesicles are detectable in macrophages even after 48 h of endocytosis. The key finding was that macrophages are able to release the internalized and stored PL back into the extracellular space[49]. Inhibition of lysosomal hydrolases with leupeptin reduced PL degradation, enhanced sorting of PL into the storage vesicles, and increased PL secretion. Thus, stabilin-1 targets PL into two distinct pathways in macrophages: lysosomal degradation and a novel pathway of TGN-associated transcytosis. TGN-associated transcytosis seems to be specific for macrophages since it was not observed in CHO and Cos7 cells stably transfected with stabilin-1 (our unpublished observations). Macrophages isolated from human placental villi (Hofbauer cells) also efficiently endocytosed PL-FITC and transported into the storage vesicles[49].

Our data showed that stabilin-1 macrophages regulate extracellular PL levels not only by the uptake and degradation, but also by the release of the internalized and stored PL. Such activity of stabilin-1–positive macrophages explains differences in PL levels in maternal and fetal circulation. PL synthesis occurs only in trophoblast cells and its secretion in placenta is believed to be constitutive. During pregnancy, PL concentration reaches 10 μg/ml in maternal circulation and stays below 0.5 μg/ml in fetal circulation; however, the cellular and molecular mechanisms that define differential concentration of hPL in maternal and fetal vessels were unknown. There is no direct transfer of hPL from the maternal to the fetal circulation. PL-producing trophoblast cells directly contact with maternal circulation, while stabilin-1+ villi macrophages are located between PL-producing trophoblast layer and fetal vessels[49]. We propose that stabilin-1–positive macrophages determine the difference in PL levels between maternal and fetal circulation. The next intriguing questions are: How is transcytosis in placental macrophages controlled during the course of pregnancy? What kind of hormones, growth factors, or cell-cell contacts define how much of extracellular PL will be degraded, how much stored, and how much released?

In summary, concentration of PL stabilin-1 can transport its molecular ligands through three different vesicular pathways: (1) endocytosis of the extracellular ligand followed by its targeting to lysosomal degradation, (2) delivery of newly synthesized protein from the biosynthetic (Golgi) compartment through the TGN to the endosomal/lysosmal system, and (3) endocytosis of the extracellular ligand followed by transport through the TGN into the storage/secretory vesicles (Fig. 2). The molecular machinery that regulates the choice of specific pathways by stabilin-1 is under investigation in our laboratory. One promising novel regulatory protein acting in TGN has been recently identified and named stabilin-1–interacting synaptotagmin-like protein (SI-Syt) (our unpublished data).

## PHAGOCYTIC FUNCTION OF STABILIN-1

Our most recent collaborative study demonstrated that the homeostatic function of stabilin-1 in macrophages is not restricted to endocytosis, but also includes phagocytosis of apoptotic bodies. Stabilin-1 is recruited to the sites of recognition and engulfment of apoptotic bodies, as well as to early phagosomes[83]. Blocking stabilin-1 in macrophages with the antibody or reducing its level by lentivirus-delivered shRNA results in defective engulfment of aged red blood cells. Ectopic expression of stabilin-1 induces the binding and engulfment of aged cells in mouse fibroblast L cells. The binding and uptake of apoptotic bodies by stabilin-1 depends on phosphatidylserine (PS), which is well known as a phagocytic ligand exposed on the surface of aging cells and is also recognized by stabilin-2 during endocytosis of apoptotic cells[84]. PS directly interacts with the EGF-like domain of stabilin-1 and is sufficient for stabilin-1–mediated phagocytosis.

Apoptotic cells are formed during developmental and homeostatic tissue turnover, as well as during the course of inflammation and tumor progression. Taking into consideration that stabilin-1, but not stabilin-2, is expressed on macrophages that participate in these physiological and pathological processes, the specific contribution of stabilin-1–mediated uptake of apoptotic cells deserves intensive investigation in animal models. Intriguing questions that have to be answered in the future using genetically modified animals as well as *in vitro* loss-of-function and gain-of-function approaches are: Does uptake of apoptotic cells via stabilin-1 support immunological tolerance or tissue remodeling? and What are the intracellular signaling cascades induced by stabilin-1 phagocytosis?

## STABILIN-1 AND CELL-CELL ADHESION: REGULATION OF INFLAMMATORY CELL TRAFFICKING

The first indication for the involvement of stabilin-1 in cell-cell adhesion was obtained by the group of Jalkanen in 2003[9]. Irjala et al.[9] reported that administration of the anti–stabilin-1 (anti–CLEVER-1) antibody 3-372 reduces lymphocyte infiltration in rabbits immunized with keyhole limpet hemocyanin. The authors suggested that the anti–CLEVER-1 antibody interferes with lymphocyte entrance in both HEV and lymphatic sinusoids. The same 3-372 antibody blocked ~50% of lymphocyte binding to lymph node sections in nonstatic and static conditions. The next study from the same group[17] presented evidence for the involvement of CLEVER-1 in adhesion of malignant cells to the lymphatic endothelium. Here, blocking of binding with 3-372 antibodies resulted in 30–50% inhibition of adhesion of malignant cells to lymphatic endothelium and HEV. In the next study from the same group, Salmi et al. showed that CLEVER-1 is involved in the transmigration of peripheral blood monocytes through the lymphatic endothelium[15]. Mixture of anti-CLEVER monoclonal antibodies 3-266 and 3-372 had a 40% inhibiting effect on peripheral blood mononuclear cell (PBMC) transmigration through the human umbilical vein endothelial cell (HUVEC) monolayer.

Most recently, in our collaborative work, we blocked stabilin-1 with both 1.26 antibody raised against mouse stabilin-1 as well as α-CLEVER-1 antibody 3-372 in order to test the involvement of stabilin-1 in cellular interactions during transmigration of immune cells[85]. CLEVER-1/stabilin-1 mediated migration of T and B lymphocytes to the draining lymph nodes *in vivo*. Moreover, stabilin-1–blocking antibodies efficiently inhibited peritonitis in mice by decreasing the entrance of granulocytes by 50%, while migration of monocytes and lymphocytes into the inflamed peritoneum was prevented almost completely. However, involvement of stabilin-1 in cell-cell contacts remains to be controversial due to its questionable expression on the surface of ECs[11]. It is necessary to identify the surface determinants on immune cells and tumor cells that interact with stabilin-1 and are essential for transmigration.

## CONCLUSION AND PERSPECTIVES

Stabilin-1, with its unusual ligand repertoire, is involved in complex physiological clearance processes. Moreover, in macrophages, stabilin-1 links two different processes: uptake and degradation of unwanted-self molecules and routing of endogenous and exogenous endocytosed ligands to the secretion pathways. As a consequence, stabilin-1 can contribute to the regulation of the secretion repertoire of extracellular mediators.

Evidence accumulates indicating involvement of stabilin-1 in direct cell-cell contacts important for the transmigration of immune cells and transformed cells. The basic biological issue that needs intensive investigation is molecular mechanism of the cross-talk between uptake, intracellular sorting, and adhesive events mediated by stabilin-1. How can the presence of extracellular stabilin-1 ligands affect its function as a cell adhesive molecule, will adhesive events recruit stabilin-1 to the cell surface and deplete it from the sorting pathways? Since new findings indicate overexpression of stabilin-1 in various pathologies, the next issues to explore are which stabilin-1 functions can contribute to the pathology and which are protective and can be used for therapy. Taking into consideration the complexity of stabilin-1 functions, investigation of stabilin-1–dependent immunological effects *in vivo* in the animal knock-out models, as well as mathematical modeling of stabilin-1–mediated processes in primary human cells, is essential in order to predict the outcome of its activities in human disorders.

## ACKNOWLEDGMENTS

I thank Dr. Alexei Gratchev for critical reading of the manuscript.

## References

1. Goerdt, S., Walsh, L.J., Murphy, G.F., and Pober, J.S. (1991) Identification of a novel high molecular weight protein preferentially expressed by sinusoidal endothelial cells in normal human tissues. *J. Cell Biol.* **113,** 1425–1437.
2. Goerdt, S., Bhardwaj, R., and Sorg, C. (1993) Inducible expression of MS-1 high-molecular-weight protein by endothelial cells of continuous origin and by dendritic cells/macrophages in vivo and in vitro. *Am. J. Pathol.* **142,** 1409–1422.
3. Kodelja, V., Muller, C., Tenorio, S., Schebesch, C., Orfanos, C.E., and Goerdt, S. (1997) Differences in angiogenic potential of classically vs alternatively activated macrophages. *Immunobiology* **197,** 478–493.
4. Politz, O., Gratchev, A., McCourt, P.A., Schledzewski, K., Guillot, P., Johansson, S., Svineng, G., Franke, P., Kannicht, C., Kzhyshkowska, J., Longati, P., Velten, F.W., Johansson, S., and Goerdt, S. (2002) Stabilin-1 and -2 constitute a novel family of fasciclin-like hyaluronan receptor homologues. *Biochem. J.* **362,** 155–164.
5. McCourt, P.A., Smedsrod, B.H., Melkko, J., and Johansson, S. (1999) Characterization of a hyaluronan receptor on rat sinusoidal liver endothelial cells and its functional relationship to scavenger receptors. *Hepatology* **30,** 1276–1286.
6. Kzhyshkowska, J., Gratchev, A., Martens, J.H., Pervushina, O., Mamidi, S., Johansson, S., Schledzewski, K., Hansen, B., He, X., Tang, J., Nakayama, K., and Goerdt, S. (2004) Stabilin-1 localizes to endosomes and the trans-Golgi network in human macrophages and interacts with GGA adaptors. *J. Leukoc. Biol.* **76,** 1151–1161.
7. Tamura, Y., Adachi, H., Osuga, J., Ohashi, K., Yahagi, N., Sekiya, M., Okazaki, H., Tomita, S., Iizuka, Y., Shimano, H., Nagai, R., Kimura, S., Tsujimoto, M., and Ishibashi, S. (2003) FEEL-1 and FEEL-2 are endocytic receptors for advanced glycation end products. *J. Biol. Chem.* **278,** 12613–12617.
8. Adachi, H. and Tsujimoto, M. (2002) FEEL-1, a novel scavenger receptor with in vitro bacteria-binding and angiogenesis-modulating activities. *J. Biol. Chem.* **277,** 34264–34270.
9. Irjala, H., Elima, K., Johansson, E.L., Merinen, M., Kontula, K., Alanen, K., Grenman, R., Salmi, M., and Jalkanen, S. (2003) The same endothelial receptor controls lymphocyte traffic both in vascular and lymphatic vessels. *Eur. J. Immunol.* **33,** 815–824.
10. Kzhyshkowska, J., Gratchev, A., and Goerdt, S. (2006) Stabilin-1, a homeostatic scavenger receptor with multiple functions. *J. Cell. Mol. Med.* **10,** 635–649.
11. Prevo, R., Banerji, S., Ni, J., and Jackson, D.G. (2004) Rapid plasma membrane-endosomal trafficking of the lymph node sinus and high endothelial venule scavenger receptor/homing receptor stabilin-1 (FEEL-1/CLEVER-1). *J. Biol. Chem.* **279,** 52580–52592.
12. Hansen, B., Longati, P., Elvevold, K., Nedredal, G.I., Schledzewski, K., Olsen, R., Falkowski, M., Kzhyshkowska, J., Carlsson, F., Johansson, S., Smedsrod, B., Goerdt, S., Johansson, S., and McCourt, P. (2005) Stabilin-1 and stabilin-2 are both directed into the early endocytic pathway in hepatic sinusoidal endothelium via interactions with clathrin/AP-2, independent of ligand binding. *Exp. Cell Res.* **303,** 160–173.
13. Martens, J.H., Kzhyshkowska, J., Falkowski-Hansen, M., Schledzewski, K., Gratchev, A., Mansmann, U., Schmuttermaier, C., Dippel, E., Koenen, W., Riedel, F., Sankala, M., Tryggvason, K., Kobzik, L., Moldenhauer, G., Arnold, B., and Goerdt, S. (2006) Differential expression of a gene signature for scavenger/lectin receptors by endothelial cells and macrophages in human lymph node sinuses, the primary sites of regional metastasis. *J. Pathol.* **208,** 574–589.
14. Qian, H., Johansson, S., McCourt, P., Smedsrod, B., Ekblom, M., and Johansson, S. (2009) Stabilins are expressed in bone marrow sinusoidal endothelial cells and mediate scavenging and cell adhesive functions. *Biochem. Biophys. Res. Commun.* **390,** 883–886.
15. Salmi, M., Koskinen, K., Henttinen, T., Elima, K., and Jalkanen, S. (2004) CLEVER-1 mediates lymphocyte transmigration through vascular and lymphatic endothelium. *Blood* **104,** 3849–3857.
16. Walsh, L.J., Goerdt, S., Pober, J.S., Sueki, H., and Murphy, G.F. (1991) MS-1 sinusoidal endothelial antigen is expressed by factor XIIIa+, HLA-DR+ dermal perivascular dendritic cells. *Lab. Invest*. **65,** 732–741.
17. Irjala, H., Alanen, K., Grenman, R., Heikkila, P., Joensuu, H., and Jalkanen, S. (2003) Mannose receptor (MR) and common lymphatic endothelial and vascular endothelial receptor (CLEVER)-1 direct the binding of cancer cells to the lymph vessel endothelium. *Cancer Res.* **63,** 4671–4676.
18. Goerdt, S., Politz, O., Schledzewski, K., Birk, R., Gratchev, A., Guillot, P., Hakiy, N., Klemke, C.D., Dippel, E., Kodelja, V., and Orfanos, C.E. (1999) Alternative versus classical activation of macrophages. *Pathobiology* **67,** 222–226.
19. Goerdt, S.and Orfanos, C.E. (1999) Other functions, other genes: alternative activation of antigen-presenting cells. *Immunity* **10,** 137–142.

20. Schledzewski, K., Falkowski, M., Moldenhauer, G., Metharom, P., Kzhyshkowska, J., Ganss, R., Demory, A., Falkowska-Hansen, B., Kurzen, H., Ugurel, S., Geginat, G., Arnold, B., and Goerdt, S. (2006) Lymphatic endothelium-specific hyaluronan receptor LYVE-1 is expressed by stabilin-1(+), F4/80(+), CD11b(+) macrophages in malignant tumours and wound healing tissue in vivo and in bone marrow cultures in vitro: implications for the assessment of lymphangiogenesis. *J. Pathol.* **209,** 67–77.
21. Schmieder, A., Schledzewski, K., Michel, J., Tuckermann, J.P., Tome, L., Sticht, C., Gkaniatsou, C., Nicolay, J.P., Demory, A., Faulhaber, J., Kzhyshkowska, J., Geraud, C., and Goerdt, S. (2010) Synergistic activation by p38MAPK and glucocorticoid signaling mediates induction of M2-like tumor-associated macrophages expressing the novel CD20 homolog MS4A8A. *Int. J. Cancer*. [Epub ahead of print]
22. Moos, V., Schmidt, C., Geelhaar, A., Kunkel, D., Allers, K., Schinnerling, K., Loddenkemper, C., Fenollar, F., Moter, A., Raoult, D., Ignatius, R., and Schneider, T. (2010) Impaired immune functions of monocytes and macrophages in Whipple's disease. *Gastroenterology* **138,** 210–220.
23. Mosig, S., Rennert, K., Krause, S., Kzhyshkowska, J., Neunubel, K., Heller, R., and Funke, H. (2009) Different functions of monocyte subsets in familial hypercholesterolemia: potential function of CD14+ CD16+ monocytes in detoxification of oxidized LDL. *FASEB J.* **23,** 866–874.
24. Soutar, A.K. and Naoumova, R.P. (2007) Mechanisms of disease: genetic causes of familial hypercholesterolemia. *Nat. Clin. Pract. Cardiovasc. Med.* **4,** 214–225.
25. Bonifacino, J.S. (2004) The GGA proteins: adaptors on the move. *Nat. Rev. Mol. Cell Biol.* **5,** 23–32.
26. Zhang, J., Gratchev, A., Riabov, V., Mamidi, S., Schmuttermaier, C., Krusell, L., Kremmer, E., Workman, G., Sage, E.H., Jalkanen, S., Goerdt, S., and Kzhyshkowska, J. (2009) A novel GGA-binding site is required for intracellular sorting mediated by stabilin-1. *Mol. Cell. Biol.* **29,** 6097–6105.
27. Ghosh, P., Dahms, N.M., and Kornfeld, S. (2003) Mannose 6-phosphate receptors: new twists in the tale. *Nat. Rev. Mol. Cell Biol.* **4,** 202–212.
28. Adachi, H. and Tsujimoto, M. (2010) Adaptor protein sorting nexin 17 interacts with the scavenger receptor FEEL-1/stabilin-1 and modulates its expression on the cell surface. *Biochim. Biophys. Acta* **1803,** 553–563.
29. Peiser, L., Mukhopadhyay, S., and Gordon, S. (2002) Scavenger receptors in innate immunity. *Curr. Opin. Immunol.* **14,** 123–128.
30. Gordon, S. (2002) Pattern recognition receptors: doubling up for the innate immune response. *Cell* **111,** 927–930.
31. Areschoug, T. and Gordon, S. (2009) Scavenger receptors: role in innate immunity and microbial pathogenesis. *Cell. Microbiol.* **11,** 1160–1169.
32. Greaves, D.R. and Gordon, S. (2005) Thematic review series: the immune system and atherogenesis. Recent insights into the biology of macrophage scavenger receptors. *J. Lipid Res.* **46,** 11–20.
33. Krieger, M. and Stern, D.M. (2001) Series introduction: multiligand receptors and human disease. *J. Clin. Invest*. **108,** 645–647.
34. Kzhyshkowska, J. and Krusell, L. (2009) Cross-talk between endocytic clearance and secretion in macrophages. *Immunobiology* **214,** 576–593.
35. Kzhyshkowska, J., Gratchev, A., Brundiers, H., Mamidi, S., Krusell, L., and Goerdt, S. (2005) Phosphatidylinositide 3-kinase activity is required for stabilin-1-mediated endosomal transport of acLDL. *Immunobiology* **210,** 161–173.
36. Maxfield, F.R. and McGraw, T.E. (2004) Endocytic recycling. *Nat. Rev. Mol. Cell Biol.* **5,** 121–132.
37. Kzhyshkowska, J., Workman, G., Cardo-Vila, M., Arap, W., Pasqualini, R., Gratchev, A., Krusell, L., Goerdt, S., and Sage, E.H. (2006) Novel function of alternatively activated macrophages: stabilin-1-mediated clearance of SPARC. *J. Immunol.* **176,** 5825–5832.
38. Brekken, R.A. and Sage, E.H. (2001) SPARC, a matricellular protein: at the crossroads of cell-matrix communication. *Matrix Biol.* **19,** 816–827.
39. Framson, P.E. and Sage, E.H. (2004) SPARC and tumor growth: where the seed meets the soil? *J. Cell. Biochem.* **92,** 679–690.
40. Arnold, S.A. and Brekken, R.A. (2009) SPARC: a matricellular regulator of tumorigenesis. *J. Cell Commun. Signal.* **3**, 255–273.
41. Hsiao, Y.H., Lien, H.C., Hwa, H.L., Kuo, W.H., Chang, K.J., and Hsieh, F.J. (2010) SPARC (osteonectin) in breast tumors of different histologic types and its role in the outcome of invasive ductal carcinoma. *Breast J.* **16,** 305–308.
42. Kos, K. and Wilding, J.P. (2010) SPARC: a key player in the pathologies associated with obesity and diabetes. *Nat. Rev. Endocrinol.* **6,** 225–235.
43. Bradshaw, A.D. and Sage, E.H. (2001) SPARC, a matricellular protein that functions in cellular differentiation and tissue response to injury. *J. Clin. Invest.* **107,** 1049–1054.
44. Murphy-Ullrich, J.E. (2001) The de-adhesive activity of matricellular proteins: is intermediate cell adhesion an adaptive state? *J. Clin. Invest.* **107,** 785–790.
45. Bradshaw, A.D. (2009) The role of SPARC in extracellular matrix assembly. *J. Cell Commun. Signal.* **3,** 239–246.
46. Koblinski, J.E., Kaplan-Singer, B.R., VanOsdol, S.J., Wu, M., Engbring, J.A., Wang, S., Goldsmith, C.M., Piper, J.T., Vostal, J.G., Harms, J.F., Welch, D.R., and Kleinman, H.K. (2005) Endogenous osteonectin/SPARC/BM-40 expression inhibits MDA-MB-231 breast cancer cell metastasis. *Cancer Res.* **65,** 7370–7377.

47. Alvarez, M.J., Prada, F., Salvatierra, E., Bravo, A.I., Lutzky, V.P., Carbone, C., Pitossi, F.J., Chuluyan, H.E., and Podhajcer, O.L. (2005) Secreted protein acidic and rich in cysteine produced by human melanoma cells modulates polymorphonuclear leukocyte recruitment and antitumor cytotoxic capacity. *Cancer Res.* **65,** 5123–5132.
48. Gordon, S. and Martinez, F.O. (2010) Alternative activation of macrophages: mechanism and functions. *Immunity* **32,** 593–604.
49. Kzhyshkowska, J., Gratchev, A., Schmuttermaier, C., Brundiers, H., Krusell, L., Mamidi, S., Zhang, J., Workman, G., Sage, E.H., Anderle, C., Sedlmayr, P., and Goerdt, S. (2008) Alternatively activated macrophages regulate extracellular levels of the hormone placental lactogen via receptor-mediated uptake and transcytosis. *J. Immunol.* **180,** 3028–3037.
50. Anthony, R.V., Fanning, M.D., Limesand, S.W., and Liang, R. (1998) Placental lactogen and growth hormone: regulation and action. In *Endocrinology of Pregnancy*. Bazer, F.W., Ed. Humana Press, Totawa, NJ. pp. 461–490.
51. Falkowski, M., Schledzewski, K., Hansen, B., and Goerdt, S. (2003) Expression of stabilin-2, a novel fasciclin-like hyaluronan receptor protein, in murine sinusoidal endothelia, avascular tissues, and at solid/liquid interfaces. *Histochem. Cell Biol.* **120,** 361–369.
52. Zhou, B., McGary, C.T., Weigel, J.A., Saxena, A., and Weigel, P.H. (2003) Purification and molecular identification of the human hyaluronan receptor for endocytosis. *Glycobiology* **13,** 339–349.
53. Harris, E.N., Baggenstoss, B.A., and Weigel, P.H. (2009) Rat and human HARE/stabilin-2 are clearance receptors for high- and low-molecular-weight heparins. *Am. J. Physiol. Gastrointest. Liver Physiol.* **296,** G1191–G1199.
54. Harris, E.N. and Weigel, P.H. (2008) The ligand-binding profile of HARE: hyaluronan and chondroitin sulfates A, C, and D bind to overlapping sites distinct from the sites for heparin, acetylated low-density lipoprotein, dermatan sulfate, and CS-E. *Glycobiology* **18,** 638–648.
55. Harris, E.N., Weigel, J.A., and Weigel, P.H. (2008) The human hyaluronan receptor for endocytosis (HARE/Stabilin-2) is a systemic clearance receptor for heparin. *J. Biol. Chem.* **283,** 17341–17350.
56. Politz, O., Kodelja, V., Guillot, P., Orfanos, C.E., and Goerdt, S. (2000) Pseudoexons and regulatory elements in the genomic sequence of the beta-chemokine, alternative macrophage activation-associated CC-chemokine (AMAC)-1. *Cytokine* **12,** 120–126.
57. Kzhyshkowska, J., Mamidi, S., Gratchev, A., Kremmer, E., Schmuttermaier, C., Krusell, L., Haus, G., Utikal, J., Schledzewski, K., Scholtze, J., and Goerdt, S. (2006) Novel stabilin-1 interacting chitinase-like protein (SI-CLP) is up-regulated in alternatively activated macrophages and secreted via lysosomal pathway. *Blood* **107,** 3221–3228.
58. Hollak, C.E., van Weely, S., van Oers, M.H., and Aerts, J.M. (1994) Marked elevation of plasma chitotriosidase activity. A novel hallmark of Gaucher disease. *J. Clin. Invest.* **93,** 1288–1292.
59. Renkema, G.H., Boot, R.G., Muijsers, A.O., Donker-Koopman, W.E., and Aerts, J.M. (1995) Purification and characterization of human chitotriosidase, a novel member of the chitinase family of proteins. *J. Biol. Chem*. **270,** 2198–2202.
60. Boven, L.A., van Meurs, M., Boot, R.G., Mehta, A., Boon, L., Aerts, J.M., and Laman, J.D. (2004) Gaucher cells demonstrate a distinct macrophage phenotype and resemble alternatively activated macrophages. *Am. J. Clin. Pathol.* **122,** 359–369.
61. Boot, R.G., Renkema, G.H., Strijland, A., van Zonneveld, A.J., and Aerts, J.M. (1995) Cloning of a cDNA encoding chitotriosidase, a human chitinase produced by macrophages. *J. Biol. Chem*. **270,** 26252–26256.
62. Boot, R.G., Blommaart, E.F., Swart, E., Ghauharali-van der Vlugt, K., Bijl, N., Moe, C., Place, A., and Aerts, J.M. (2001) Identification of a novel acidic mammalian chitinase distinct from chitotriosidase. *J. Biol. Chem*. **276,** 6770–6778.
63. Renkema, G.H., Boot, R.G., Au, F.L., Donker-Koopman, W.E., Strijland, A., Muijsers, A.O., Hrebicek, M., and Aerts, J.M. (1998) Chitotriosidase, a chitinase, and the 39-kDa human cartilage glycoprotein, a chitin-binding lectin, are homologues of family 18 glycosyl hydrolases secreted by human macrophages. *Eur. J. Biochem.* **251,** 504–509.
64. Zhu, Z., Zheng, T., Homer, R.J., Kim, Y.K., Chen, N.Y., Cohn, L., Hamid, Q., and Elias, J.A. (2004) Acidic mammalian chitinase in asthmatic Th2 inflammation and IL-13 pathway activation. *Science* **304,** 1678–1682.
65. Hogdall, E.V., Johansen, J.S., Kjaer, S.K., Price, P.A., Christensen, L., Blaakaer, J., Bock, J.E., Glud, E., and Hogdall, C.K. (2003) High plasma YKL-40 level in patients with ovarian cancer stage III is related to shorter survival. *Oncol. Rep.* **10,** 1535–1538.
66. Cintin, C., Johansen, J.S., Christensen, I.J., Price, P.A., Sorensen, S., and Nielsen, H.J. (1999) Serum YKL-40 and colorectal cancer. *Br. J. Cancer* **79,** 1494–1499.
67. Cintin, C., Johansen, J.S., Christensen, I.J., Price, P.A., Sorensen, S., and Nielsen, H.J. (2002) High serum YKL-40 level after surgery for colorectal carcinoma is related to short survival. *Cancer* **95,** 267–274.
68. Jensen, B.V., Johansen, J.S., and Price, P.A. (2003) High levels of serum HER-2/neu and YKL-40 independently reflect aggressiveness of metastatic breast cancer. *Clin. Cancer Res.* **9,** 4423–4434.
69. Dupont, J., Tanwar, M.K., Thaler, H.T., Fleisher, M., Kauff, N., Hensley, M.L., Sabbatini, P., Anderson, S., Aghajanian, C., Holland, E.C., and Spriggs, D.R. (2004) Early detection and prognosis of ovarian cancer using serum YKL-40. *J. Clin. Oncol*. **22,** 3330–3339.
70. Tanwar, M.K., Gilbert, M.R., and Holland, E.C. (2002) Gene expression microarray analysis reveals YKL-40 to be a potential serum marker for malignant character in human glioma. *Cancer Res.* **62,** 4364–4368.

71. Steenbakkers, P.G., Baeten, D., Rovers, E., Veys, E.M., Rijnders, A.W., Meijerink, J., De Keyser, F., and Boots, A.M. (2003) Localization of MHC class II/human cartilage glycoprotein-39 complexes in synovia of rheumatoid arthritis patients using complex-specific monoclonal antibodies. *J. Immunol.* **170,** 5719–5727.
72. Vind, I., Johansen, J.S., Price, P.A., and Munkholm, P. (2003) Serum YKL-40, a potential new marker of disease activity in patients with inflammatory bowel disease. *Scand. J. Gastroenterol.* **38,** 599–605.
73. Tran, A., Benzaken, S., Saint-Paul, M.C., Guzman-Granier, E., Hastier, P., Pradier, C., Barjoan, E.M., Demuth, N., Longo, F., and Rampal, P. (2000) Chondrex (YKL-40), a potential new serum fibrosis marker in patients with alcoholic liver disease. *Eur. J. Gastroenterol. Hepatol.* **12,** 989–993.
74. Saitou, Y., Shiraki, K., Yamanaka, Y., Yamaguchi, Y., Kawakita, T., Yamamoto, N., Sugimoto, K., Murata, K., and Nakano, T. (2005) Noninvasive estimation of liver fibrosis and response to interferon therapy by a serum fibrogenesis marker, YKL-40, in patients with HCV-associated liver disease. *World J. Gastroenterol.* **11,** 476–481.
75. La Montagna, G., D'Angelo, S., and Valentini, G. (2003) Cross-sectional evaluation of YKL-40 serum concentrations in patients with systemic sclerosis. Relationship with clinical and serological aspects of disease. *J. Rheumatol.* **30,** 2147–2151.
76. Owhashi, M., Arita, H., and Hayai, N. (2000) Identification of a novel eosinophil chemotactic cytokine (ECF-L) as a chitinase family protein. *J. Biol. Chem.* **275,** 1279–1286.
77. Recklies, A.D., White, C., and Ling, H. (2002) The chitinase 3-like protein human cartilage glycoprotein 39 (HC-gp39) stimulates proliferation of human connective-tissue cells and activates both extracellular signal-regulated kinase- and protein kinase B-mediated signalling pathways. *Biochem. J.* **365,** 119–126.
78. Ling, H. and Recklies, A.D. (2004) The chitinase 3-like protein human cartilage glycoprotein 39 inhibits cellular responses to the inflammatory cytokines interleukin-1 and tumour necrosis factor-alpha. *Biochem. J.* **380,** 651–659.
79. Nishikawa, K.C. and Millis, A.J. (2003) gp38k (CHI3L1) is a novel adhesion and migration factor for vascular cells. *Exp. Cell Res.* **287,** 79–87.
80. Oba, Y., Chung, H.Y., Choi, S.J., and Roodman, G.D. (2003) Eosinophil chemotactic factor-L (ECF-L): a novel osteoclast stimulating factor. *J. Bone Miner. Res.* **18,** 1332–1341.
81. Areshkov, P.A. and Kavsan, V.M. (2010) Chitinase 3-like protein 2 (CHI3L2, YKL-39) activates phosphorylation of extracellular signal-regulated kinases ERK1/ERK2 in human embryonic kidney (HEK293) and human glioblastoma (U87 MG) cells. *Tsitol. Genet*. **44,** 3–9.
82. Renkema, G.H., Boot, R.G., Strijland, A., Donker-Koopman, W.E., van den Berg, M., Muijsers, A.O., and Aerts, J.M. (1997) Synthesis, sorting, and processing into distinct isoforms of human macrophage chitotriosidase. *Eur. J. Biochem.* **244,** 279–285.
83. Park, S.Y., Jung, M.Y., Lee, S.J., Kang, K.B., Gratchev, A., Riabov, V., Kzhyshkowska, J., and Kim, I.S. (2009) Stabilin-1 mediates phosphatidylserine-dependent clearance of cell corpses in alternatively activated macrophages. *J. Cell Sci.* **122,** 3365–3373.
84. Park, S.Y., Jung, M.Y., Kim, H.J., Lee, S.J., Kim, S.Y., Lee, B.H., Kwon, T.H., Park, R.W., and Kim, I.S. (2008) Rapid cell corpse clearance by stabilin-2, a membrane phosphatidylserine receptor. *Cell Death Differ*. **15,** 192–201.
85. Karikoski, M., Irjala, H., Maksimow, M., Miiluniemi, M., Granfors, K., Hernesniemi, S., Elima, K., Moldenhauer, G., Schledzewski, K., Kzhyshkowska, J., Goerdt, S., Salmi, M., and Jalkanen, S. (2009) Clever-1/Stabilin-1 regulates lymphocyte migration within lymphatics and leukocyte entrance to sites of inflammation. *Eur. J. Immunol.* **39,** 3477–3487.
86. Kzhyshkowska, J., Marciniak-Czochra, A., and Gratchev, A. (2008) Perspectives of mathematical modelling for understanding of intracellular signalling and vesicular trafficking in macrophages. *Immunobiology* **212,** 813–825.
